# The Impact of Dementia's Affiliate Stigma on the Mental Health of Relatives: A Cross Section Survey

**DOI:** 10.3389/fpsyg.2021.789105

**Published:** 2022-01-20

**Authors:** Pauline Van den Bossche, Birgitte Schoenmakers

**Affiliations:** ^1^Faculty of Medicine, KU Leuven, Leuven, Belgium; ^2^Department of Public Health and Primary Care, KU Leuven, Leuven, Belgium

**Keywords:** dementia, family care, mental health, primary care, taboo

## Abstract

**Objective:**

To determine the impact of the affiliate stigma on mental well-being of relatives caring for a person with dementia.

**Design:**

The study was conducted in a cross sectional design.

**Setting:**

The study was conducted in a public setting, addressing relatives caring for a person with dementia.

**Participants:**

Participants were relatives of patients with a formal diagnosis of dementia. Relatives were defined as caring or living closely to a patient. Participants were recruited with the help of care and welfare organizations.

**Outcome Measures:**

The main outcome measure was the impact of the affiliate stigma on mental well-being of caring relatives.

**Results:**

228 participants fully completed the survey. Women, relatives with a higher education and partners experienced more impact of the affiliate stigma on mental well-being than man, relatives with a lower education and relatives with another relationship to the person with dementia (resp. F-ratio = 15.67; *p* = 0.0001; F-ratio = 2.5865; *p* = 0.0381; F-ratio = 3.1131; *p* = 0.0099). The duration of dementia and the age of the caregiver had a clear significant effect on affiliate stigma (F-ratio = 4.9104; *p* = 0.0083) (F-ratio = 6.5515, *p* = 0.0112).

**Conclusion:**

This study revealed that caregiver related features are predicting the presence of an affiliate stigma. Interventions to prevent or reduce the impact of this stigma might focus on these groups. Education about dementia and the impact on patients, relatives and the broader social context might alter the affiliate stigma surrounding dementia.

## Introduction

Dementia is the collective name given to a group of symptoms that indicates a significant decline in a person's level of cognitive functioning. This syndrome has a global prevalence of about 7% in the population aged 65 years or older (Prince et al., 2013). Currently, more than 48 million people suffer from dementia worldwide. Without effective treatment, the World Health Organization estimates that this prevalence will continue to rise to 131 million people by 2050 (Alzheimer, 2016; Gale et al., 2018; Wolters and Ikram, 2018).

In addition to the decline in cognitive functioning, persons with dementia also experience a significant loss in social, occupational and/or domestic functioning. This makes them dependent on persons in their environment to carry out daily activities. This dependence puts a great physical, emotional and financial burden on the relatives of persons with dementia (Schoenmakers et al., 2010a; Chow et al., 2018). The assumptions made in our society about persons with dementia are often based on stereotypes. This stereotypical presentation is influenced by the later stages of the syndrome, where the person with dementia is fully care dependent (Van Gorp and Vercruysse, 2012). The result of these negative assumptions is that self-stigmatization occurs in both persons with dementia and their relatives, which can cause them feeling insecure in their dealing with the person with dementia (Greenwood et al., 2018; Jeong et al., 2020; Rewerska-Juśko and Rejdak, 2020). In certain cases, stigma negatively influences the opportunities of a timely diagnosis of dementia (Dubois et al., 2016). The affiliation stigma appears relatively independently of the type of dementia and seems rather related to other features and characteristics of patients, caregivers and community (Greenwood et al., 2018; Rewerska-Juśko and Rejdak, 2020).

In 2008, the WHO released a report defining the concept of stigma. With stigma, a distinction is made between the person who is stigmatized and others who attribute negative characteristics to the stigmatized person. In the event that stigma is present in the context of mental illness, it often results in social exclusion and discrimination, which in turn creates an additional emotional burden (Europe WHOROF, 2008). In addition to the emotional burden present in the stigmatized individual, several studies report that the family caregivers and relatives of individuals with mental illness also often experience stigma. This form of stigma present among family caregivers and relatives has been described as “courtesy stigma” or “associative stigma” (Goffman, 1963; Mehta and Farina, 1988). These terms point to the discrimination and prejudice that people may experience because they are associated with individuals who belong to a stigmatized group. In addition to “courtesy stigma,” other studies focused on the internalization of stigma that lives among family caregivers and relatives of individuals with mental illness. This internalization was described as “affiliate stigma.” The term “affiliate stigma” refers to the negative feelings that relatives of stigmatized individuals develop toward themselves, because they perceive the associative stigma that prevails in society. These negative feelings can cause attitude changes toward the person with dementia. Making less contact with the person with dementia, concealing the association with them from the public or generally less engaging in social contact are some examples (Mak and Cheung, 2008).

To date, only a limited number of studies have investigated the affiliate stigma associated with dementia. The purpose of this study is to identify the affiliate stigma present among the relatives of individuals with dementia and its effect on their mental well-being.

## Methods

### Design

This study examined the relationship between the affiliate stigma experienced by relatives of persons with dementia and the impact on their mental health well-being.

Data for this study were collected cross-sectionally over a 4-month period from August 2020 to November 2020. All participants were informed of the anonymity of the data handling and the purpose of the study prior to starting the survey.

### Setting and Participants

Survey participants were relatives of patients with a formal diagnosis of dementia (diagnosed by a physician). No further specification of type or stage of dementia was required since caregiver and contextual characteristics are single predictors of care burden and affiliate stigma (Schoenmakers et al., 2010a; Rewerska-Juśko and Rejdak, 2020). Relatives were defined as caring or living closely to a patient without restriction in time spent caring or relationship with the care receiver (self-appointed carer). Participants were recruited with the help of care and welfare organizations. Participants who did not have a close relative with dementia, formal or professional caregivers and participants who did not complete the survey were excluded from the study or from further data analysis.

To calculate the sample size we started from a convenience sample of 500 participants to achieve a confidence interval between 1.5 and 2.5 for the score on mental well-being. The desired sample consisted of 217 participants.

### Collection of Data

The survey was presented on a secured platform (server of the university KU Leuven) using Qualtrics Software. Study data were collected anonymously *via* a survey distributed through social media with a special focus on communities of family caregivers, relatives, and acquaintances of persons with dementia. The Expertise Centre Dementia Flanders was involved in the recruitment process. In addition, the survey was also shared through the site of a home nursing group that cares for patients with dementia and their relatives. Before starting the survey, the purpose of the study was explained. Surveys that were not completed in full were excluded from further processing.

The survey consisted of three parts: the first part contained questions about the background features of the participants (age, gender, and highest education degree), their relationship with the person with dementia, and how long ago the patient was diagnosed with dementia.

The questions in the second part of the survey were taken from the Affiliate Stigma Scale (ASS) (Mak and Cheung, 2008). The Affiliate Stigma Scale was originally developed to gauge the self-stigmatization that occurs among family caregivers, relatives, or acquaintances of patients with mental illness. The term “mental illness” was replaced with “dementia” in the Affiliate Stigma Scale. The scale consists of 22 questions that test for the internalization of stigma present in relatives. The scale addresses the cognitive, affective, and behavioral domain. The cognitive and the affective component contain 7 items and the behavioral component contains eight items. Participants answered all 22 items using a 4-point Likert scale ranging from “1 = Totally disagree” to “4 = Totally agree.” From these 22 items, the mean affiliate stigma score was calculated, which was a measure of reported stigma. The higher the mean score on the Affiliate Stigma Scale, the higher the internalized stigma. There is no cut off score.

The third section of the survey mapped the mental well-being of the participating relatives. This survey was composed based upon items from the Patient Health Questionnaire-9 and the 20-item Center for Epidemiologic Studies Depression Screening (Williams et al., 1999; Siu et al., 2016). Ten indicators of perceived mental well-being were included in the survey: helpless, failed, ashamed, concentration troubles, sleeping troubles, tiredness (little energy), less appetite, sadness, anxiety, anger. Answer options were distributed on a 4-point Likert scale ranging from “1 = Never” to “4 = Always.” A mean score was calculated from the values reported on these ten items and named the “mood score” in further analysis. The higher this mean score, the lower the mental well-being. There was no cut off score.

### Data Analysis

The collected data were processed via Excel for descriptive analyses and from there imported into SAS9.4 for multivariate analyses using the General Linear Model procedure (GLM) with estimated effects.

The hypothesis assumed a significant effect of the affiliate stigma reported by the relatives of persons with dementia on their mental well-being (mood-score). The GLM-procedure was used to calculate the effect (by the estimated effect measure) of the affiliate stigma on mental well-being including all background features (independent variables). Second, the impact of the background features on affiliate stigma was analyzed, also by GLM-procedure (F-distribution).

### Ethical Consideration

The authors assert that all procedures contributing to this work comply with the ethical standards of the relevant national and institutional committees on human experimentation and with the Helsinki Declaration of 1975, as revised in 2008 (Puri et al., 2009). All procedures involving human subjects/patients were approved by the Medical Ethical Board of the University Hospitals of Leuven under the number MP015226 (May 2019) prior to data collection. Participants were comprehensively informed about the study and explicitly agreed to participate. By a simple opting out button, participants could withdraw from further participation.

### Patient and Public Involvement

There was no patient or public involvement in the conception or dissemination of this study.

## Results

Over a 4-month period, 504 relatives started the survey. During data processing, 40 participants were excluded because they did not have a relative with dementia. In addition, another 236 participants were excluded due to early termination of the survey. 228 participants were included in the final data analysis.

The mean age of the participants was 42.92 years, with a standard deviation (SD) of 15.75 years (Table 1). Most of the participants were women (193, 84.65%). Half of the participants earned a bachelor's degree (114, 50.00%) and about one-third earned a high school diploma (70, 30.70%). For the majority of those who completed the survey, the nearest person with dementia was a parent (97, 42.54%) or grandparent (73, 32.02%). Dementia was diagnosed more than 1 year ago in the majority of cases (199, 87.28%) (Table 1).

**Table 1:** Background features of the participants.

**Feature**	**Number**	**%**
**Demographic features** ***n*** **=** **228**		
Age in years	42.92 (average)	Stand dev (15.75)
**Sex**		
Male	35	15.35%
Female	193	84.65%
**Education degree**		
No degree	5	2.19%
Highschool	70	30.70%
Bachelor	114	50.00%
Master	35	15.35%
PhD	4	1.75%
**Relation to the patient**		
Partner	14	6.14%
Sibling	7	3.07%
Parent	97	42.54%
Grandparent	73	32.02%
Aunt/uncle	8	3.51%
Friend/neighbor	24	10.53%
No data	5	2.19%
**Time since diagnosis**		
<6 months	8	3.51%
6 months to 1 year	19	8.33%
> 1 year	199	87.28%
No data	2	0.88%

The mean affiliate stigma score of the participants was 1.63 [95% CI (1.57, 1.70)]. The mean mood-score of participants was 1.83 [95% CI (1.76, 1.89)]. The estimated effect of the affiliate stigma score on mental well-being, considering account age, gender, degree, relationship with the patient and duration of dementia was 0.66 [95% CI (0.55, 0.76), *p* <0.001] (Table 2).

**Table 2:** Impact of affiliate stigma score on mental well-being, adjusted for age, gender, diploma, relationship with the person with dementia and time since diagnosis.

**Term**	**Estimate**	**Std error**	**t ratio**	**Prob>|t|**	**Lower 95%**	**Upper 95%**
Intercept	0.8655918	0.262209	3.30	0.0011^*^	0.3485889	1.3825947
Affiliate Stigma Score mean	0.65527	0.051272	12.78	<0.0001^*^	0.5541761	0.756364
Age	0.0007379	0.002343	0.31	0.7531	−0.003882	0.0053576
Sex (M)	−0.261222	0.065981	−3.96	0.0001^*^	−0.391318	−0.131126
Degree (Bachelor diploma)	0.1786387	0.164229	1.09	0.2780	−0.145175	0.5024525
Degree (PhD)	0.2081076	0.239022	0.87	0.3850	−0.263176	0.679391
Degree (Master diploma)	0.20349	0.172063	1.18	0.2383	−0.135769	0.542749
Degree (highschool)	0.3304696	0.164059	2.01	0.0453	0.0069919	0.6539474
Relation (sibling)	−0.341489	0.164282	−2.08	0.0389	−0.665407	−0.01757
Relation (grandparents)	−0.357473	0.130205	−2.75	0.0066	−0.614201	−0.100745
Relation (parent)	−0.318028	0.104772	−3.04	0.0027	−0.524608	−0.111447
Relation (aunt/uncle)	−0.600482	0.159235	−3.77	0.0002^*^	−0.914449	−0.286515
Relation (Friend/neighbor)	−0.346818	0.128372	−2.70	0.0075	−0.599932	−0.093704
Time since diagnosis (<6 months)	−0.037392	0.128639	−0.29	0.7716	−0.291032	0.2162485
Time since diagnosis (1 year−6 months)	0.0206053	0.088116	0.23	0.8153	−0.153134	0.1943451

*N = 228*.

* *p <0.01*.

*Estimated effect as contrast with: Sex: female*.

*Education degree: no degree*.

*Relationship: partners*.

*Time since diagnosis: > 1 year*.

There was a significantly different impact of affiliate stigma on mental well-being between men and women (F-ratio = 15.67; *p* <0.001) where women experienced a higher impact [0.26, 95% CI (0.13, 0.39), *p* <0.001]. Education also predicted the impact of affiliate stigma on mental well-being (F-ratio = 2.58; *p* = 0.038). There was a significant difference between individuals who did not obtain a DIPLOMA and those with a high school diploma experiencing less impact of [0.33, 95% CI (0.001, 0.65)] (*p* = 0.045) (Table 2).

The relationship between participants and the person with dementia affected the impact of affiliate stigma on mental well-being (F-ratio = 3.1131; *p* = 0.01). The partners experienced the largest impact of affiliate stigma on mental well-being as compared to all other relationships {mean mood score of 2.01 [95% CI (1.77, 2.25)]}. This impact significantly differs from the children's mean mood score of 1.69 [95% CI (1.54, 1.84), *p* = 0.00]. The aunts and uncles of persons with dementia experienced the lowest impact of affiliate stigma on their mental well-being and presented with a mean mood score of 1.41 [95% CI (1.14, 1.68)], with an estimated difference of 0.60 [95% CI (0.29, 0.91)] as compared to the partners (*p* = 0.0002) (Tables 2, 3).

**Table 3:** Distribution of background features for affiliate stigma.

**Source**	**Nparm**	**DF**	**Sum of squares**	**F ratio**	**Prob > F**
Age	1	1	1.4436932	6.5515	0.0112
Sex	1	1	0.0535956	0.2432	0.6224
Degree	4	4	1.6324794	1.8521	0.1203
Relation	5	5	2.4800308	2.2509	0.0507
Time since diagnosis	2	2	2.1641102	4.9104	0.0083^*^

*N = 228*.

* *p <0.01*.

*Nparm, non-parametric test for multivariate data; DF, degrees of freedom*.

The impact of the demographic factors on the affiliate stigma score was analyzed secondarily. The relationship with the patient was a nearly significant predictor of the affiliate stigma (F-ratio = 2.251; *p* = 0.051) (Table 3). This analysis showed that the duration of dementia and the age of the care had a significant effect on affiliate stigma (F-ratio = 4.910; *p* = 0.008) (F-ratio = 6.5515, *p* = 0.011). Relatives of patients who had received the formal diagnosis of dementia for more than 1 year, presented with a mean affiliate stigma score of 1.65 [95% CI (1.48,1.82)], which was significantly higher {0.51 [95% CI (0.17, 0.85)]} than in relatives of patients who received the diagnosis less than 6 months ago (*p* = 0.0033). Older caregivers seemed also suffering more form affiliate stigma than their younger colleagues do [−0.08, 95%CI (−0.001 to 0.014)] (Table 4).

**Table 4:** Impact of the demographic factors on the affiliate stigma score.

**Term**	**Estimate**	**Std error**	**t ratio**	**Prob>|t|**	**Lower 95%**	**Upper 95%**
Intercept	1.350816	0.239517	5.64	<0.0001^*^	0.8785706	1.8230614
Age	−0.008061	0.003149	−2.56	0.0112^*^	−0.01427	−0.001852
Sex (M)	−0.044408	0.090046	−0.49	0.6224	−0.221949	0.1331326
Degree (Bachelor diploma)	−0.004354	0.074761	−0.06	0.9536	−0.151757	0.1430488
Degree (PhD)	−0.105318	0.247695	−0.43	0.6711	−0.593688	0.3830525
Degree (Master diploma)	−0.290234	0.223106	−1.30	0.1948	−0.730122	0.1496545
Degree (highschool)	0.2014181	0.102802	1.96	0.0514	−0.001273	0.4041095
Relation (sibling)	0.2289657	0.220934	1.04	0.3013	−0.206641	0.6645726
Relation (grandparents)	−0.016039	0.126772	−0.13	0.8994	−0.265992	0.2339129
Relation (parent)	0.1901151	0.114073	1.67	0.0971	−0.034797	0.4150277
Relation (aunt/uncle)	0.4922047	0.171877	2.86	0.0046^*^	0.1533216	0.8310877
Relation (Friend/neighbor)	0.2829005	0.195482	1.45	0.1494	−0.102523	0.6683242
Time since diagnosis (<6 months)	0.3700052	0.2028	1.82	0.0695	−0.029849	0.7698589
Time since diagnosis (1 year−6 months)	0.5118241	0.171968	2.98	0.0033^*^	0.1727613	0.8508869

* *p < 0.01*.

*Estimated effect as contrast with: Sex: female*.

*Education degree: no degree*.

*Relationship: partners*.

*Time since diagnosis: >1 year*.

## Discussion

Literature investigating affiliate stigma in the context of dementia is limited to date. This research shows that the affiliate stigma surrounding dementia negatively affects the mental well-being of close relatives. Female caregivers and partners are particularly affected. Furthermore, from this study, it appears that the longer the diagnosis of dementia exists and the older the caregiver, the higher the experience of the affiliate stigma.

Prior studies to date focused on the affiliate stigma in family caregivers of persons with dementia, whereas this study has also included other relatives. Caregivers who appoint themselves as a caregiver are considered as formal caregivers independently of their relationship with the care receiver or the time spent caring (Schoenmakers et al., 2010a, 2011).

This study shows that the impact of the affiliate stigma on mental well-being is higher in female relatives, which is in line with the findings in previous research (Kahn et al., 2016). More women than men take up the role of family caregiver and women often adopt different coping strategies than men (Schoenmakers et al., 2009; Werner et al., 2012). Coping strategies are more determined by caregivers characteristics than by care characteristics; female caregivers generally use a more emotional coping strategy while male caregivers tend to cope in a more problem solving way (Schoenmakers et al., 2009, 2010a). This might explain why woman suffer more form the affiliate stigma. Above, being a family caregiver implies a physical and emotional burden, which has an additional negative effect on mental well-being (Schulz and Martire, 2004; Papastavrou et al., 2007; Ask et al., 2014).

When considering the relationships with the person with dementia, it appears that partners and children report the highest impact of affiliate stigma on mental well-being, which was confirmed in previous research (Werner et al., 2012; Kahn et al., 2016). Partners generally are permanently on care duty and therefore more burdened (Schoenmakers et al., 2010a). Second, the intra-relation and intra-familial role switch is a source of stress for the caring partner and children (Schoenmakers et al., 2010a).

While previous research suggests that lower educational attainment is associated with a higher effect of stigma on mental well-being, this study reveals the opposite (Krajewski et al., 2013; Werner and AboJabel, 2020). In our study, the small number of participants without an education degree might affect the findings. On the other hand, being highly educated might also reduce the impact of feelings of shame as “it (dementia) appears to happen in the best families” (Schoenmakers et al., 2010a).

The results from this study indicate that if the formal diagnosis of dementia exists for a longer period, relatives report a higher affiliate stigma. An explanation for this might be that these relatives already experience a high burden, reinforcing the impact of negative feelings and in this case of the affiliate stigma (Schoenmakers et al., 2009). The relationship between reported affiliate stigma and duration since the formal diagnosis of dementia needs further investigation in the future.

This study has several limitations. First, this study included relatives of patients with all different types of dementia. Since dementia is defined as a syndrome with a broad range of etiologies, the presentation of behavioral changes of persons with dementia also differs. These different presentations might influence the onset of affiliate stigma in relatives. Second, this study was conducted in a selected population (recruited through care and welfare organizations), whereby the different groups were not equally represented. Above, participating relatives were already supported by professionals or at least “help seeking,” since they were recruited through organizations. Therefore, the resilience of these participants might differ from relatives caring without support (Schoenmakers et al., 2010b). Third, the items used to survey mental well-being were selected from existing surveys. We included items explicitly referring to mental well-being: sleep, cognitive functioning, social functioning, appetite, mood status. This selection was drawn by consensus among the researcher and we did not perform a validation of this survey. We thereby only focussed on the negative of mental well-being.

The strength of the study is the relatively large study population as compared to other studies. The use of the self-rating Affiliate Stigma Scale for this population is relatively unique. The affiliate stigma on dementia is well known but poorly studied. The use of the self-rating Affiliate Stigma Scale is therefore a strength. This scale has been reported in previous studies to have a broad scope, as it can be efficiently applied to both family caregivers and other family members of different levels of education (Saffari et al., 2019). We particularly opted not to use the Family Stigma in Alzheimer's Disease Scale (FS-ADS) as this is instrument focusses on Alzheimer's Disease while our premise is that the type of dementia does not play a major role in the presentation of affiliate stigma.

## Conclusion

Only few studies are available that investigate affiliate stigma and its effect on mental well-being. From this study, we have learned that some groups are more prone to impact of the affiliate stigma on well-being. Women, partners and relatives with a higher education need particular attention and support to lower the impact of the affiliate stigma on mental well-being. In addition, the longer the diagnosis of dementia exists, the higher the affiliate stigma. Education about dementia and the impact on patients, relatives and the broader social context might alter the affiliate stigma surrounding dementia.

## Data Availability Statement

The original contributions presented in the study are included in the article/supplementary materials, further inquiries can be directed to the corresponding author.

## Ethics Statement

The studies involving human participants were reviewed and approved by Medical Ethical Board of the University Hospitals of Leuven under the number MP015226. The patients/participants provided their written informed consent to participate in this study.

## Author Contributions

PV and BS: conception and design of the research, analysis of data, and revision the article. PV: acquisition of data and drafting the article. All authors read and approved the final manuscript.

## Conflict of Interest

The authors declare that the research was conducted in the absence of any commercial or financial relationships that could be construed as a potential conflict of interest.

## Publisher's Note

All claims expressed in this article are solely those of the authors and do not necessarily represent those of their affiliated organizations, or those of the publisher, the editors and the reviewers. Any product that may be evaluated in this article, or claim that may be made by its manufacturer, is not guaranteed or endorsed by the publisher.
